# Therapeutic Notes

**Published:** 1896-06-06

**Authors:** 


### THERAPEUTIC NOTES.

### FERMENTS IN DIABETIC TREATMENT.

About eight years ago V. Mering and Minkowski showed that excision of the pancreas in dogs caused permanent diabetes, and that result did not ensue if a small piece of the gland was left *in situ*, or was transplanted under the skin of the abdomen. More recently these results have been confirmed by Shabad,[1] and a large number of cases have been put on record of diabetes associated with disease of the pancreas in man. For these and other reasons it has been thought that the pancreas by a process of so-called internal-secretion shed into the blood a ferment which was capable of destroying an excess of sugar, and that in diabetes the presence of sugar in the blood and urine was due to an absence of this protective process, either from disease of the pancreas, or from inhibition of its normal activity. From the fact that diabetes, due to the extirpation of the pancreas, could be successfully treated by engrafting a piece of pancreas into the abdominal cavity, it was suggested that the same result might follow if the pancreas were given as food by the mouth, and in the case of dogs Ausset[2] has shown that after extirpation of the pancreas the diabetic symptoms disappeared if a calf's pancreas were given mixed up with the ordinary food. Further, he showed that these excellent results lasted as long as the pancreas was administered, but ceased when this treatment was discontinued. The same author also gives us details of a case in which he tried the same method of treatment in a human diabetic patient, who was passing thirty-eight grams of sugar per diem with more than double the normal elimination of chlorides and phosphates. On the second day of treatment he noticed a decrease of thirty-four grams of sugar eliminated in the twenty-four hours, and a return to the normal condition as regards chlorides and phosphates. On the ninth day the sugar had entirely disappeared, and the urine remained normal for over a month. In this case we have no note as to the supposed character of the diabetes, or its probable cause; that is to say, whether of pancreatic or other origin.

More recently we have the contribution of Lissere[3] on the same subject. His experiences were drawn from a considerable number of cases treated by the same method. His conclusions are as follows: If pancreatic extract be given diabetics by the mouth (1) the quantity of sugar in the urine is always decreased; (2) that this diminution in the total quantity of urine is accompanied by a corresponding diminution in the total quantity of the urine passed; (3) that there is a general improvement in the condition of the patient, hunger and thirst being diminished; (4) that on leaving off the treatment the symptoms return as before.

He recommends that the extract be made as follows: A fresh pancreas should be taken from a bullock, minced and macerated with an equal quantity of water containing 10 per cent. sodium chloride and 3 per cent. bicarbonate of soda. Of this extract 80 to 100 grams should be given twice daily.

On somewhat similar lines, Cassaët[4] has tried the effect of giving large quantities of ordinary brewers' yeast. In spite of considerable flatulence, putrid diarrhœa, and other unpleasant symptoms, this treatment seems to have modified the quantity of sugar in several instances; and the general condition of the patient as regards weight, &c., apparently improved. These results, which require confirmation, are possibly analogous to those which follow a strictly limited carbohydrate diet, since the presence of a large quantity of yeast in the stomach and intestines would certainly destroy by fermentative processes any sugar or carbohydrate which might otherwise reach the blood. The treatment by pancreatic ferments is without doubt harmless, and in certain cases may give good results, hence, where practicable, it should be tried.

[1] Vratch, Nos. 47-49, 1892. [2] Sem. Med., August 21, 1895. [3] Medisinskoïe Obosrenïe, No. 4, 1896. [4] Sem. Med., August 21, 1895.

### SERUM INJECTION IN INFECTIOUS FEVERS.

Early last year Surgeon Kinyoun[1] (U.S. Navy) published the report of certain cases of variola treated by the injection of blood serum taken from a heifer calf vaccinated four weeks previously; fifteen centimetres of the serum was injected subcutaneously when the case came under notice, and again after the lapse of some hours. A careful study of the case led the writer to suppose that the course of the fever was distinctly modified in a favourable direction. We now have the report of some somewhat analogous cases of Dr. Baginsk,[2] in which a considerable number of scarlatina patients were treated with Marmorek's serum from the Pasteur Institute. The object in view was to combat late complications and sequelæ, such as otitis or nephritis. The experiments were carried out in November of last year, fiftyseven cases in all, from which total nine must be deducted, since, in these the treatment was insufficient or too slow. In twenty-seven cases the temperature declined immediately after the injection—a result probably, but not necessarily, due to the injection. With angina or swollen glands, the inflammation disappeared in a few days. In a second group of fifteen cases the treatment appeared quite inefficacious, and some few very severe cases succumbed rapidly, in spite of the treatment, even when maximum doses were employed. In other cases less severe, swelling of the glands continued until it was necessary to incise them and let out the pus. In a third group of five cases in which the serum was employed with the object of preventing contingent complications, the results were entirely satisfactory, it being found unnecessary to repeat the initial injection. To sum up, the mortality of all cases treated on these lines was 14 per cent., as compared to 22 to 24 per cent. the previous mortality before the adoption of the method. The difference Dr. Bagensky regards as too small to form a favourable opinion of the method. The results show, however, that there is no danger to be feared from the injection, and that experience may teach better use to be made of the principle.

[1] Phil. Med. News, Feb., 1895. [2] Rev. d. Therapeut., April 1st, 189

